# Clinical factors associated with discontinuation of ts/bDMARDs in rheumatic patients from the BIOBADASER III registry

**DOI:** 10.1038/s41598-021-90442-w

**Published:** 2021-05-27

**Authors:** A. Prior-Español, C. Sánchez-Piedra, J. Campos, F. J. Manero, C. Pérez-García, C. Bohórquez, N. Busquets-Pérez, J. M. Blanco-Madrigal, C. Díaz-Torne, F. Sánchez-Alonso, L. Mateo, S. Holgado-Pérez

**Affiliations:** 1 Rheumatology Unit, Hospital Universitari Germans Trias I Pujol, Carretera del Canyet, s/n, 08916 Badalona, Barcelona, Spain; 2 Research Unit, Spanish Society of Rheumatology, Madrid, Spain; 3 Rheumatology Unit, Hospital Puerta de Hierro, Madrid, Spain; 4 Rheumatology Unit, Hospital Universitario Miguel Servet, Zaragoza, Spain; 5 Rheumatology Unit, Hospital del Mar, Barcelona, Spain; 6 Rheumatology Unit, Hospital Universitario Príncipe de Asturias, Alcalá de Henares, Spain; 7 Rheumatology Unit, Hospital General de Granollers, Barcelona, Spain; 8 Rheumatology Unit, Hospital de Basurto, Vizcaya, Spain; 9 Hospital Santa Creu I Sant Pau, Barcelona, Spain

**Keywords:** Rheumatoid arthritis, Spondyloarthritis, Ankylosing spondylitis, Psoriatic arthritis

## Abstract

Biologic and targeted synthetic disease-modifying antirheumatic drugs (ts/bDMARDs) play a pivotal role in the treatment of rheumatoid arthritis (RA), psoriatic arthritis (PsA), and ankylosing spondylitis (AS). Persistence of therapy provides an index of a drug’s overall effectiveness. The objective of the study was to identify factors associated with discontinuation of ts/bDMARDs in a real-world dataset. The study population comprised patients diagnosed with RA, PsA, and AS included in the BIOBADASER registry for whom follow-up data were available until November 2019. Patient features and treatment data were included in the analysis. The Kaplan–Meier method was used to study survival of the different drugs according to the reason for discontinuation. Factors associated with discontinuation were studied using Cox regression models and bivariate and multivariate analyses. P values of less than 0.05 were regarded as statistically significant. The study population comprised 4,752 patients who received a total of 8,377 drugs, of which 4,411 (52.65%) were discontinued. The Kaplan–Meier curves showed that survival for first-line treatment was greater in all 3 groups (p < 0.001). Patients with RA had a greater risk of discontinuation if they were younger (HR, 0.99; 95% CI 0.99–1.00), if they were receiving anti-TNFα agents (HR, 0.61; 95% CI 0.54–0.70), and if they had more comorbid conditions (HR, 1.09; 95% CI 1.00–1.17). Patients with PsA had a higher risk if they were women (HR, 1.36; 95% CI 1.15–1.62) and if they were receiving other ts/bDMARDs (HR, 1.29; 95% CI 1.05–1.59). In patients with AS, risk increased with age (HR, 1.01; 95% CI 1.00–1.02), as did the number of comorbid conditions (HR, 1.27; 95% CI 1.12–1.45). The factors that most affected discontinuation of ts/bDMARDs were line of treatment, age, type of drug, sex, comorbidity and the year of initiation of treatment. The association with these factors differed with each disease, except for first-line treatment, which was associated with a lower risk of discontinuation in all 3 diseases.

## Introduction

Prescription of biologic disease-modifying antirheumatic drugs (bDMARDs) and targeted synthetic DMARDs (tsDMARDs) to patients with rheumatoid arthritis (RA), psoriatic arthritis (PsA), and ankylosing spondylitis (AS) has increased considerably in recent decades. The advantages of these agents include long-term efficacy and a favorable safety profile^1,2^. Persistence, or survival, of ts/bDMARDs gives us a general idea about their effectiveness, safety, and tolerability^3^.

Various studies have analyzed drug persistence. One real-world study on factors associated with persistence of golimumab found that the retention rate of this drug was increased when it was used as the first-line biologic or concomitantly with methotrexate^4^. In the TOCERRA registry, the retention rate and efficacy of tocilizumab in monotherapy or in combination (second or successive line of treatment) were higher than and similar to, respectively, those of anti-TNFα agents combined with methotrexate^5^. The various analyses of anti-TNFα agents performed in the BIOBADASER registry have investigated changes in discontinuation patterns in recent years^6^, age as a predictor of the reason for discontinuation^7^, better persistence of anti-TNFα agents in AS than in RA^8^, survival of anti-TNFα agents after failure of a first anti-TNFα agent^9^, and safety and retention of drugs prescribed off-label^10^. Other studies have analyzed factors that may affect persistence of biologics, including age, sex, underlying disease, comorbidity, and line of treatment^11,12^.

Nevertheless, few studies have included a general evaluation of how patient clinical factors and the type of treatment affect the discontinuation rates of various drugs in different diseases. Thus, the objectives of the present study were to evaluate how the patient’s characteristics and the type of drug affect discontinuation in each of the 3 groups of patients (RA, AS, PsA) and to investigate the impact of new drugs with novel mechanisms of action in recent years.

## Patients and methods

### Study design and setting: BIOBADASER 3.0

The present study was a real-world multicenter prospective study of patients with RA, PsA, or AS initiating a bDMARD or tsDMARD, with follow-up data until November 2019. Information was obtained from BIOBADASER III, a national prospective registry of patients with rheumatic diseases treated with bDMARDs, including biosimilars, and tsDMARDs, either with approved or off-label indications.

Phase III of this registry was initiated at the end of 2015. Patients from the previous phase were able to continue if they gave their written informed consent for their data to be collected again in this phase. The objectives and methodology of the register’s successive phases have been described elsewhere^13^. The registry protocol and relevant materials are available at http://biobadaser.ser.es.

In brief, BIOBADASER 3.0 recruits patients from 28 large public hospitals throughout Spain, with an estimated national coverage of bDMARD and tsDMARD treatment in RA of nearly 25%. Patients included in the registry are followed-up prospectively and evaluated when an adverse event (AE) occurs or treatment with ts/bDMARDs is changed. Evaluations are performed to assess lack of efficacy (switching to a different drug) or toxicity (discontinuation or dose reduction) at least once a year. The full database is monitored online to assess its consistency and quality; additionally, a random sample of patients (mean per center = 12) is selected and audited in situ at all 28 centers annually.

### Population

This nested cohort comprised patients from the BIODASER registry diagnosed with RA, PsA, or AS according to the criteria of their treating rheumatologist who were prescribed a ts/bDMARD.

### Outcome variables

The data included in this analysis were as follows: (1) patient data, including sex, date of birth, diagnosis, and date of diagnosis, (2) baseline status, including comorbidities (Charlson index) and risk factors (body mass index [BMI]); and (3) data on treatment, including time on biologic treatment, line of treatment, and type of biologics (according to the therapeutic target).

Discontinuation of treatment was defined as the interruption of treatment for a period ≥ 3 months. Temporary discontinuations (i.e., those lasting < 3 months) were not included.

### Statistical analysis

Descriptive results are presented as mean and standard deviation or as numbers and percentages, as appropriate. Kaplan–Meier analysis was used to study survival of ts/bDMARDs, and various analyses were performed according to the reason for discontinuation. Factors associated with discontinuation were studied using Cox regression models. Bivariate and multivariate analyses were performed to study those factors. Statistical significance was set at p < 0.05.

All analyses were performed using Stata version 13.1 (Stata Corp., College Station, TX, USA, 2013).

All procedures and materials complied with the principles of the Declaration of Helsinki and with Spanish regulations on data protection and research. Ethical approval was granted by the Hospital Clinic of Barcelona Ethics Committee (one of the participating centers) acting as a reference committee (approval code FER-ADA-2015–01). All patients signed informed consent before register inclusion.

## Results

We included 4,752 patients (2,381 in the RA group 1,121 in the AS group, and 1,250 in the PsA group). Table 1 shows the characteristics of the patients analyzed. Women were more common in the RA group (79.71%) and men in the AS group (69.94%), whereas the sex distribution was even in the PsA group (51.12% women). Most patients (55.68%) had been diagnosed more than 5 years before initiation of their ts/bDMARD. Mean age at initiation of therapy was 51.62 ± 12.94 years. Most patients were overweight or obese (64.94%). The mean age-adjusted Charlson comorbidity index was 2.13 ± 1.45. The 4,752 patients received a total of 8,377 ts /bDMARDs with anti-TNFα drugs predominating in the 3 groups (63.47%). Etanercept was the most frequently used drug (20.01%), followed by adalimumab (18.04%). Of the 8,377 treatments included, 2,719 (32.46%) were started before or during the year 2014 and 5,658 (67.54%) after the year 2014. A total of 4,411 drugs were discontinued (52.65%), mainly owing to lack of efficacy or loss of efficacy (49.13%) or because of AEs (23.31%). The most frequently indicated concomitant drugs in the 3 groups were methotrexate (60.35%) and corticosteroids (62.9%).

**Table 1:** Baseline clinical characteristics of patients and type of treatment.

Patients	RA		AS		PsA		Total	
	n	%	n	%	n	%	n	%
Men	483	20.29	784	69.94	611	48.88	1878	39.52
Women	1898	79.71	337	30.06	639	51.12	2874	60.48
**Time since diagnosis (years)**								
< 1	187	7.85	204	18.2	182	14.56	573	12.06
1–4	761	31.96	312	27.83	460	36.8	1533	32.26
5–10	591	24.82	210	18.73	301	24.08	1102	23.19
> 10	842	35.36	395	35.24	307	24.56	1544	32.49
**BMI**								
< 19	37	2.13	12	1.41	13	1.37	62	1.75
19–25	618	35.6	301	35.29	259	27.35	1178	33.31
25–30	625	36	334	39.16	354	37.38	1313	37.13
30–35	323	18.61	157	18.41	213	22.49	693	19.6
> 35	133	7.66	49	5.74	108	11.4	290	8.2
**Treatment**								
Anti-TNFα	2278	50.83	1576	87.56	1463	69.80	5317	63.47
Etanercept	872	19.46	342	19	462	22.04	1676	20.01
Infliximab	392	8.74	340	18.89	211	10.07	943	11.26
Adalimumab	566	12.63	479	26.61	466	22.23	1511	18.04
Golimumab	221	4.93	295	16.39	213	10.16	729	8.70
Certolizumab	227	5.07	120	6.67	111	5.30	458	5.47
Other ts/bDMARDs	2203	49.16	224	12.44	633	30.20	3060	36.53
Anakinra	11	0.25	0	0	1	0.05	12	0.14
Rituximab	392	8.75	0	0	3	0.14	395	4.72
Abatacept	583	13.01	0	0	10	0.48	593	7.08
Tocilizumab	683	15.24	0	0	0	0.00	683	8.15
Sarilumab	64	1.43	0	0	0	0.00	64	0.76
Ustekinumab	0	0	12	0.67	174	8.30	186	2.22
Apremilast	0	0	1	0.06	120	5.73	121	1.44
Secukinumab	0	0	209	11.61	281	13.41	490	5.85
Ixekizumab	0	0	0	0	23	1.10	23	0.27
Tofacitinib	250	5.58	2	0.11	20	0.95	272	3.25
Baricitinib	220	4.91	0	0	1	0.05	221	2.64
**Reason for discontinuation**								
Lack of efficacy	1190	45.51	418	51.1	559	57.16	2167	49.13
Adverse event	629	24.05	182	22.25	217	22.19	1028	23.31
Pregnancy/desire to become pregnant	55	2.1	17	2.08	26	2.66	98	2.22
Patient lost	23	0.88	20	2.44	13	1.33	56	1.27
Remission	40	1.53	17	2.08	30	3.07	87	1.97
Other	479	18.32	125	15.28	97	9.92	701	15.89
Unknown	199	7.61	39	4.77	36	3.68	274	6.21
**Concomitant treatment**								
Corticosteroids	3292	75.3	212	22.53	617	49.8	4121	62.9
Methotrexate	2576	66.6	272	27.87	878	66.02	3726	60.35
Leflunomide	1177	36.29	34	3.97	357	34.03	1568	30.46
	Mean	SD	Mean	SD	Mean	SD	Mean	SD
Age at onset (years)	55.36	12.44	45.97	12.67	49.57	11.78	51.62	12.94
Age-adjusted Charlson index	2.42	1.58	1.73	1.19	1.93	1.3	2.13	1.45

*RA* rheumatoid arthritis; *AS* ankylosing spondylitis; *PsA* psoriatic arthritis; *BMI* body mass index; *SD* standard deviation.

### Kaplan–Meier survival analysis

**Figure 1:**
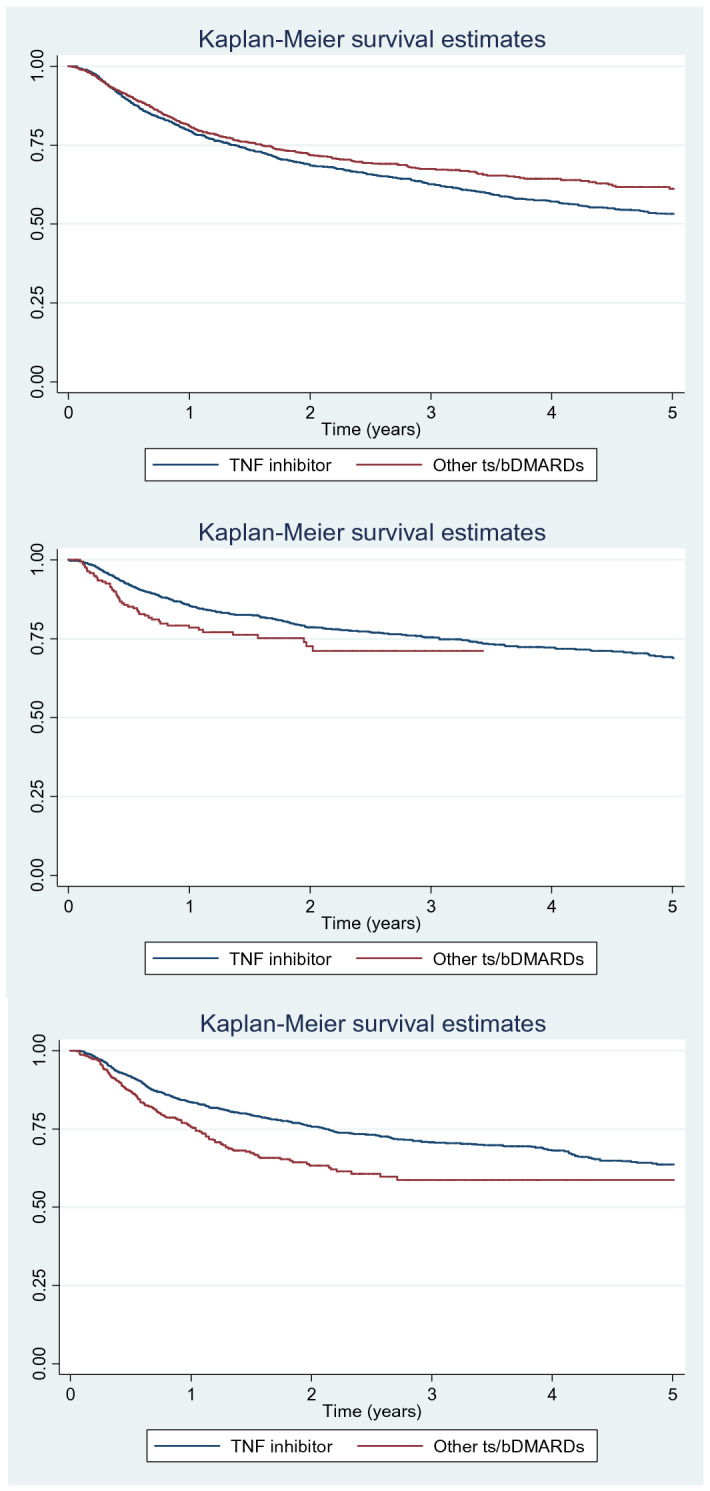
Kaplan–Meier survival curves for anti-TNFα vs. other ts/bDMARDs. (**a**) RA group (p = 0.01). (**b**) AS group (p = 0.02). (**c**) PsA group (p < 0.001).

The treatment retention curves (anti-TNFα vs. other ts/bDMARDs) in the models examining discontinuation due to lack of efficacy revealed significant differences in all 3 groups of patients. Survival of other ts/bDMARDs was greater in patients with RA (p = 0.01), whereas that of anti-TNFα drugs was greater in patients with AS (p = 0.02) and PsA (< 0.001) (Fig. 1).

**Figure 2:**
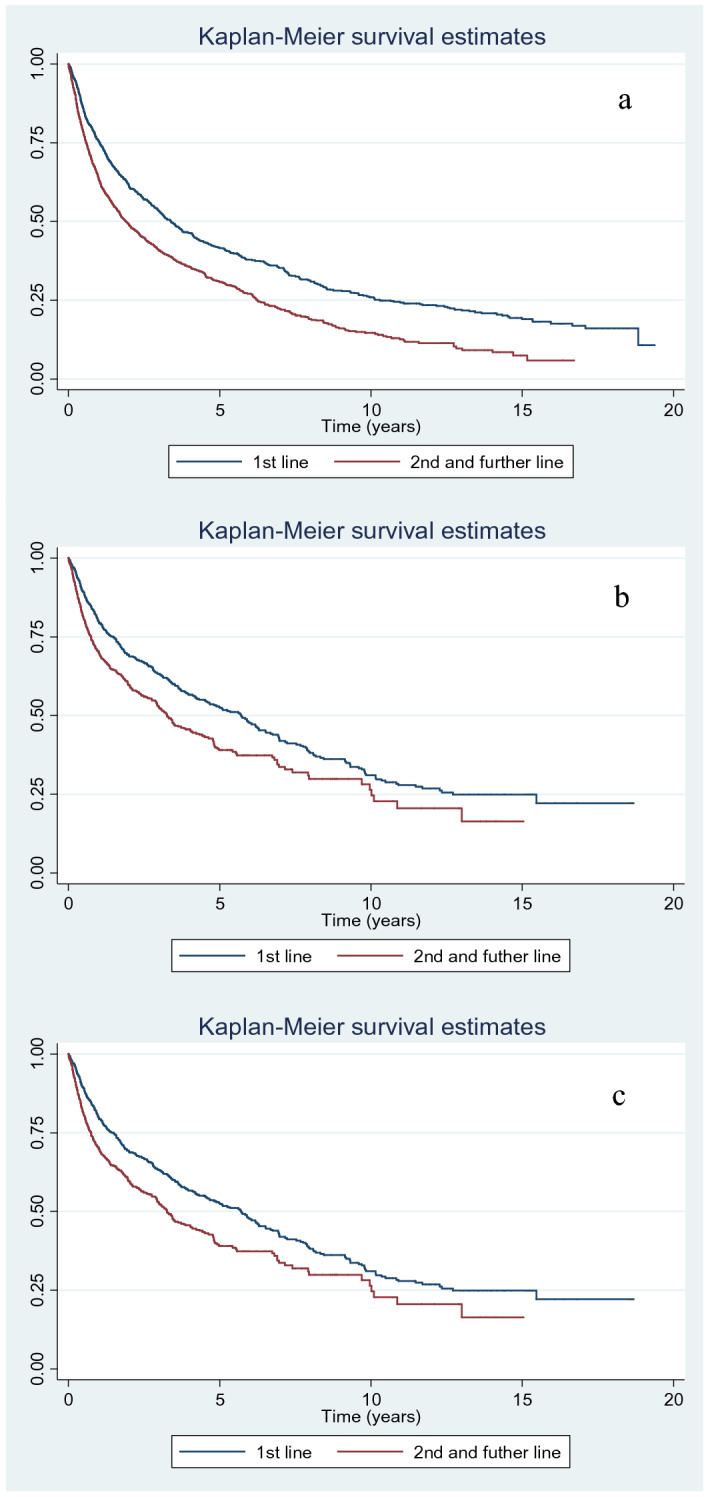
Kaplan–Meier survival curves for first-line treatment compared with second-line and successive treatments. (**a**) RA group (p < 0.001). (**b**) AS group (p < 0.001). (**c**) PsA group (p < 0.001).

The treatment retention curves comparing first-line with second-line and successive treatments revealed significant differences in all 3 groups of patients (p < 0.001), with greater survival for first-line agents (Fig. 2).

### Factors associated with treatment discontinuations in RA

Table 2 shows the results of the multivariate regression model in RA patients. In the model examining lack of efficacy, the factors associated with a greater risk of discontinuation were second and further lines of treatment (hazard ratio [HR], 1.85; 95% CI 1.62–2.12) and starting treatment after the year 2014 (HR, 1.19; 95% CI 1.05–1.36). A lower risk of discontinuation was observed for patients with older age at onset (HR, 0.99; 95% CI 0.99–1.00), longer time since diagnosis (HR, 0.99; 95% CI 0.98–0.99), and treatment with other ts/bDMARDs (HR, 0.61; 95% CI 0.54–0.70). In the model examining discontinuation because of AEs, a greater risk was observed for patients with older age at onset (HR, 1.01; 95% CI 1.00–1.02) and a higher Charlson comorbidity index (HR, 1.09; 95% CI 1.00–1.17). A lower risk of discontinuation was observed for patients starting treatment after the year 2014 (HR, 0.69; 95% CI 0.56–0.86).

**Table 2:** Factors associated with treatment discontinuation. Multivariate regression model in the group of patients with rheumatoid arthritis.

Lack of efficacy	HR	95% CI	p
Sex (ref. male)	1.14	(0.98–1.32)	0.098
Age at onset (years)	0.99	(0.99–1.00)	0.002
ts/bDMARDs (ref. anti-TNFα)	0.61	(0.54–0.70)	< 0.001
Time since diagnosis (years)	0.99	(0.98–0.99)	< 0.001
Line of treatment (ref. first line)	1.85	(1.62–2.12)	< 0.001
Starting ts/bDMARDs after the year 2014 (ref. ≤ 2014)	1.19	(1.05–1.36)	0.008

Adverse events	HR	95% CI	p
Sex (ref. male)	1.15	(0.90–1.46)	0.262
Age at onset (years)	1.01	(1.00–1.02)	0.031
Charlson comorbidity index	1.09	(1.00–1.17)	0.042
Methotrexate (ref. no methotrexate)	0.84	(0.69–1.01)	0.066
Starting ts/bDMARDs after the year 2014 (ref. ≤ 2014)	0.69	(0.56–0.86)	0.001

*HR* hazard ratio; *CI* confidence interval.

### Factors associated with treatment discontinuation in PsA

Table 3 shows the results of the multivariate regression model in patients with PsA. In the model examining lack of efficacy, a greater risk was observed in women (HR, 1.36; 95% CI 1.15–1.62), patients receiving second and further lines of treatment (HR, 1.69; 95% CI 1.41–2.03), patients who were receiving other ts/bDMARDs (HR, 1.29; 95% CI 1.05–1.59), and patients who start treatment after the year 2014 (HR, 1.29; 95% CI 1.04–1.59). In the model examining discontinuation due to AEs, women (HR, 1.92; 95% CI 1.44–2.56) and older patients (HR, 1.01; 95% CI 1.00–1.03) were at greater risk of discontinuation.

**Table 3:** Factors associated with treatment discontinuation. Multivariate regression model in the group of patients with psoriatic arthritis.

Lack of efficacy	HR	95% CI	p
Sex (ref. male)	1.36	(1.15–1.62)	< 0.001
Age at onset (years)	0.99	(0.99–1.00)	0.16
ts/bDMARDs (ref. anti-TNFα)	1.29	(1.05–1.59)	0.015
Line of treatment (ref. first line)	1.69	(1.41–2.03)	< 0.001
Starting ts/bDMARDs after the year 2014 (ref. ≤ 2014)	1.29	(1.04–1.59)	0.022

Adverse events	HR	95% CI	p
Sex (ref. male)	1.92	(1.44–2.56)	< 0.001
Age at onset (years)	1.01	(1.00–1.03)	0.016
ts/bDMARDs (ref. anti-TNFα)	0.78	(0.54–1.12)	0.183
Starting ts/bDMARDs after the year 2014 (ref. ≤ 2014)	1.16	(0.83–1.62)	0.397

*HR* hazard ratio; *CI* confidence interval.

### Factors associated with treatment discontinuation in AS

Table 4 shows the results of the multivariate regression model in patients with AS. In the model examining lack of efficacy, a greater risk was observed in older patients (HR, 1.01; 95% CI 1.00–1.02), second and further lines of treatment (HR, 2.17; 95% CI 1.67–2.83), concomitant treatment with methotrexate (HR, 1.33; 95% CI 1.02–1.75), and smoking (HR, 1.4; 95% CI 1.07–1.83). In the model examining discontinuation due to AEs, a greater risk was found in patients with more comorbid conditions (HR, 1.27; 95% CI 1.12–1.45).

**Table 4:** Factors associated with treatment discontinuation. Multivariate regression model in the group of patients with ankylosing spondylitis.

Lack of efficacy	HR	95% CI	p
Sex (ref. male)	1.12	(0.85–1.47)	0.415
Age at onset (years)	1.01	(1.00–1.02)	0.012
Line of treatment (ref. first line)	2.17	(1.67–2.83)	< 0.001
Methotrexate (ref. no methotrexate)	1.33	(1.02–1.75)	0.037
Smoker (ref. nonsmoker)	1.4	(1.07–1.83)	0.015
Starting ts/bDMARDs after the year 2014 (ref. ≤ 2014)	0.85	(0.64–1.13)	0.256

Adverse events	HR	95% CI	p
Sex (ref. male)	1.31	(0.96–1.78)	0.085
Age at onset (years)	1	(0.98–1.01)	0.764
Charlson comorbidity index	1.27	(1.12–1.45)	< 0.001
Starting ts/bDMARDs after the year 2014 (ref. ≤ 2014)	0.74	(0.53–1.01)	0.061

*HR* hazard ratio; *CI* confidence interval.

## Discussion

The present study shows that various factors affect discontinuation of treatment with bDMARDs and tsDMARDs, whether because of lack of efficacy or because of AEs. These factors do not affect the 3 rheumatic diseases studied here in the same way. Age played a role in all the groups, albeit to a different extent. Patients with RA had a greater risk of discontinuation due to lack of efficacy at an earlier age, although older patients had a greater risk of discontinuation due to AEs. The risk increased with age in patients with PsA and AS too. Anti-TNFα agents were associated with a greater risk of discontinuation in patients with RA but a lower risk in those with PsA. The risk of discontinuation was greater in women with PsA. The presence of comorbid conditions was associated with a greater risk of discontinuation in patients with RA and AS.

In both the survival analysis and the multivariate regression models, first-line treatment was associated with a lower risk of discontinuation, irrespective of the underlying disease or type of treatment received. Similar findings were previously reported in the BIODASER registry in patients receiving the anti-TNFα agents available at the time^9^. Several studies also highlight greater persistence with first-line treatments in patients with PsA^14,15^. A study performed in Spanish patients confirmed the greater rates found for retention of first-line treatments in patients with RA and AS^16^.

In the case of patients with RA, those who were younger were at a greater risk of discontinuing treatment owing to lack of efficacy but at a lower risk of discontinuing treatment owing to AEs. In contrast, in patients with AS, the risk of discontinuation owing to lack of efficacy increased with age. One previous study reported that younger patients were more likely to discontinue treatment owing to lack of efficacy and older patients owing to AEs^7^. Persistence has also been shown to be greater in older patients than in younger patients^11,12^. The broad availability of treatment options in RA could explain in part these differences in persistence according to age. An additional factor may be that the objective of treatment is to achieve optimal control of the disease as quickly as possible in younger patients with a shorter time since diagnosis. The lower number of targets for available treatment of AS implies longer maintenance of drugs in these patients.

Patients with a shorter time since diagnosis of RA had a greater risk of discontinuing treatment owing to inefficacy. This finding is controversial, given that previous studies have demonstrated that treatment with biologics is more effective the sooner it is started^17,18^. It was precisely these findings that prompted the emergence of the “treat to target” strategy^19^. The present study did not show treatment to be less effective in patients with a shorter time since diagnosis; rather, it showed that this group had a greater risk of discontinuation. In our case, these findings could arise from the fact that patients requiring treatment with ts/bDMARDs at an early stage of their disease are usually more complex, with poor prognostic factors. Consequently, our treatment objectives were more ambitious and demanding, thus leading to more frequent changes in treatment.

The risk of discontinuation was greater in patients with RA receiving anti-TNFα. Data reported elsewhere show that the discontinuation rate for anti-TNFα gradually increased during the first year of treatment. However, the authors drew no comparisons with other groups^6^. Consistent with our findings, various studies of patients with RA reported a higher retention rate for other ts/bDMARDs (abatacept, tocilizumab) than for anti-TNFα drugs^20,21^. However, other publications showed that the discontinuation rate was lower in patients receiving anti-TNFα agents as their first-line treatment^3,22^.

The risk of discontinuing treatment because of AEs in patients with RA and AS was greater for those who had a higher Charlson comorbidity index. In line with these results, other studies have reported that retention of biologics was poorer in patients with a higher comorbidity index^12,22,23^.

Women with PsA were at greater risk of discontinuing the drug owing to both lack of efficacy and AEs. Consistent with these findings, the DANBIO registry revealed a greater persistence of anti-TNFα agents in men with PsA^24^. Nevertheless, results reported elsewhere did not indicate statistically significant differences in the retention of drugs according to sex or type of drug used^25^*.* These findings differ from those of the present study, where patients who received other ts/bDMARDs drugs were at greater risk of discontinuation owing to lack of efficacy. Other publications have reported a high persistence of treatment with anti-TNFα drugs in patients with PsA^14,26^. Nevertheless, few real-world studies compare the efficacy and retention of anti-TNFα drugs with those of other ts/bDMARDs drugs in these patients^14^, possibly because other ts/bDMARDs drugs are newer. Data have been published from 2 head-to-head clinical trials comparing 2 drugs with different mechanisms of action: adalimumab and secukinumab^27^ and adalimumab and ixekizumab^28^. In the first, secukinumab was not shown to be more efficacious than adalimumab in terms of the ACR20 at week 52, although retention was greater for secukinumab^27^. In the second, ixekizumab was equally effective as adalimumab for control of arthritis^28^. In the case of patients with PsA, it was observed that when treatment was discontinued because of an AE, the association with age at onset was significant, and the risk of discontinuation increased by 2% with each year of age. It was recently reported that age increased the incidence of the first AE and that this is the main risk factor for onset of AE in patients with RA, PsA, and AS^29^. A real-life study that compared retention of anti-TNFα agents in patients with juvenile idiopathic arthritis (< 16 years) and patients with adult-onset disease found that adults more frequently discontinued treatment owing to lack of efficacy and AEs (severe infection and neoplasms), although both groups had similar retention rates at 10 years^30^.

Patients with RA and PsA have a greater risk of discontinuing treatment owing to inefficacy if they initiated treatment during 2015–2019 compared with those who started treatment in previous years. Given the greater availability and variety of therapeutic targets in these diseases in recent years, there is a tendency to switch treatment. In line with these findings, other studies have reported that, over the years, the tendency to make more changes in treatment is increasing^22,31^. In the case of discontinuation owing to adverse events, patients with RA had a lower risk of discontinuing owing to adverse events during 2015–2019 with respect to previous years. During the early years of biologics, the lack of experience may have led these agents to be discontinued quickly when an adverse event occurred. However, over the years, knowledge of the safety profile of the drugs has improved^1,32,33^.

Our study has both strengths and limitations. We analyzed data from a national registry with abundant real-world, clinical practice data and followed patients over a long period. We also included recent persistence data that reflect the impact of the drugs aimed at new therapeutic targets. Our main limitation is that some variables that could be of interest in this type of analysis were not collected appropriately; for example, disease activity indexes and the patient’s weight on discontinuing treatment and starting a new treatment were not homogeneously collected for all patients. The study is also limited by the way treatment with rituximab was recorded, since initiation and discontinuation of each cycle or dose of treatment are recorded separately. The reason for discontinuation of cycles of rituximab is usually recorded as “other” in most cases. Therefore, the analyses performed in the present study did not take into account some of the cases of discontinuation of rituximab where it was not specified that the reason for discontinuation was the lack of efficacy or adverse events. Nevertheless, considering the number of treatments with rituximab included in the analysis (< 5%), the impact of this observation on our findings can be considered minimal.

## Conclusion

The discontinuation of ts/bDMARDs analyzed in the present study seems to be affected by various factors. First-line treatment was associated with a lower risk of discontinuing treatment in all 3 diseases studied. Age, sex, type of drug, the presence of comorbid conditions and the year of initiation of treatment were associated with differences in the retention rate of the treatments studied. The association was different for each of the diseases studied. The availability of more treatment options in some of the diseases studied may have changed trends in the use of drugs and in the factors associated with discontinuation rates.

## Ethics approval

All procedures and materials complied with the principles of the Declaration of Helsinki and with Spanish regulations on data protection and research. Ethical approval was granted by the Hospital Clinic of Barcelona Ethics Committee (one of the participating centers) acting as a reference committee (approval code FER-ADA-2015–01).

## Consent to participate

All patients gave their written informed consent to be included in the BIOBADASER registry. Informed consent included consent for subsequent analysis, such as the present analysis. Patients’ information was managed as anonymized aggregated data and, as approved by the Clinical Research Committee; specific informed consent for this analysis was not required.

## Consent for publication

All of the authors consent to the publication of the manuscript. This manuscript has not been submitted for publication to any other journal, and the data it reports have not been published elsewhere and are not being evaluated by other journals.

## Data Availability

The data that support the findings of this study are available from Spanish Society of Rheumatology but restrictions apply to the availability of these data, which were used under license for the current study, and so are not publicly available. Data are however available from the authors upon reasonable request and with permission of Spanish Society of Rheumatology.
